# Toward a Molecular Profile of Self-Representation

**DOI:** 10.3389/fnhum.2016.00602

**Published:** 2016-11-28

**Authors:** Victòria Brugada-Ramentol, Gonzalo G. de Polavieja, Ángel-Carlos Román

**Affiliations:** Collective Behavior Lab, Champalimaud Research, Fundaçao ChampalimaudLisboa, Portugal

**Keywords:** self-representation, OMICS techniques, sense of agency, sense of ownership, proteomics

## Abstract

Feeling embodiment over our body or body part has a major role in the understanding of the self and control of self-actions. Even though it is crucial in our daily life, embodiment is not an homogenous phenotype across population, as quantified by implicit and explicit measures (i.e., neuroimaging or self-reports). Studies have shown differences in neuropathological conditions compared to healthy controls, but also across healthy individuals. We discuss examples of self-perception differences, and the molecular origin of embodiment, focusing on clinical cases, during the first and second section. We then discuss two important questions in this molecular-to-embodiment relationship: (i) which are the molecular levels (and their associated techniques) that can be relevant to embodiment, and (ii) which are the most adequate experiments to correlate molecular profiles and embodiment quantification across individuals. Potential answers for both questions will be outlined during the third and fourth sections, respectively, in order to design a framework to study the molecular profile of body embodiment.

## Embodiment and variability

Sense of embodiment refers to the feeling of owning and controlling a body (Kilteni et al., 2012), leading to the believe that it is the own body. This contributes to generate the representation of the bodily-self in the brain. Self-representation has two distinct subcomponents, sense of ownership (i.e., attributing a body or body part to the self) and sense of agency (i.e., having sense of control over the action). The former arises from the combination of multiple information sources, such as visual or tactile (Gallagher, 2000), the latter arises when the efferent copy of an intention of an action and its actual sensory outcome match (Wolpert and Miall, 1996; Gallagher, 2000).

Since Botvinick and Cohen developed the Rubber Hand Illusion paradigm (Botvinick and Cohen, 1998), the study of self-representation has grown interest in the field of cognitive sciences. The way the bodily-self is represented becomes crucial for the interaction of the individual with the environment and with other individuals. Moreover, this relationship is not unidirectional, as this self-representation is updated with the information obtained from the interaction with external sources. By means of the sensory organs, the brain receives multisensory information that is used to update and change self- and other-representation constantly. Due to this plasticity the process is malleable experimentally, thus generating embodiment over fake body parts or full bodies (Botvinick and Cohen, 1998; Lenggenhager et al., 2007; Slater et al., 2008). The effect of these changes has not only been reported behaviorally, but also through neuroimaging studies using fMRI. It has been shown that changes in the representation of the self caused experimentally (i.e., rubber hand illusion, or full body swap illusion) cause changes in activity in multisensory brain areas, when compared to conditions in which there is no reported illusion by the subjects (Ehrsson et al., 2005; Petkova et al., 2011). The implication of these areas (i.e., premotor cortex, intraparietal cortex, and putamen) suggests the importance of the multisensory integration on the feeling of ownership.

More importantly, embodiment has been shown to be affected by neurodevelopmental disorders (e.g., autism spectrum disorders) and in several psychiatric and neurological conditions (see a summary in Table 1). Autism spectrum disorder patients have been shown to be less susceptible to feel ownership of a rubber hand (Palmer et al., 2015). Also, other experimental paradigms reported an altered sense of embodiment over the own body in the case of autistic patients (Conson et al., 2015), as their findings suggest that these patients rely less on one's own information to infer states over another person or external object. On the other hand, schizophrenic patients perceive other-generated actions as their own in a bimanual interference task, thus presenting an increased sense of agency (Garbarini et al., 2016). This could be due to an alteration in the comparison between estimation and actual sensory feedback of the action. Another case is the one of hemiplegic patients that can feel ownership over another person performing an action, even when the arm is in the position of the limb over which they have no control (Garbarini et al., 2015). Moreover, some dementias (e.g., frontotemporal dementia) can also affect patient's sense of ownership (measured by the rubber hand illusion) and sense of agency (using a test for the attribution of an action) (Downey et al., 2014). Interestingly, Alzheimer's patients seem to have a similar self-perception than their healthy counterparts, but with defects in the attribution of self- vs. non-self memories (Bond et al., 2016). Finally, anorexia nervosa causes profound alterations in body image perception (Keizer et al., 2011, 2012) and patients with Medically Unexplained Symptoms (MUS) also show defects in body representation (Miles et al., 2011).

**Table 1:** **Summary of the neuropathological conditions affecting self-perception and embodiment**.

**Condition**	**Phenotype**	**Genes altered**	**References**
Schizophrenia	Increased sense of agency in a bimanual interference task	More than 100, not specifically associated to embodiment	Schizophrenia Working Group of the Psychiatric Genomics Consortium, 2014; Sekar et al., 2016
Autism Spectrum Disorders	Decreased sense of ownership in rubber hand illusion	More than 100, not specifically associated to embodiment	Schizophrenia Working Group of the Psychiatric Genomics Consortium, 2014
Hemiplegia	Increased sense of ownership over an external arm	ATP1A3	Heinzen et al., 2012
Frontotemporal dementia	Increased sense of ownership in rubber hand illusion and tactile discrimination	C9ORF72, MAPT	Downey et al., 2014
Alzheimer	Deficit for recognizing self voice	APP, PSEN1	Bond et al., 2016
Anorexia Nervosa	Altered tactile estimation	ESRRA, HDAC4	Cui et al., 2013
Medically Unexplained Symptom (MUS)	Reduced sense of ownership in rubber hand illusion	NA	

Nonetheless, variability in phenotypes related to embodiment can also be seen in healthy subjects, which, for example, have different levels of reported sense of ownership and agency when embodying a virtual limb. Moreover, the response to modulations to the embodied virtual limb is also different depending on the participant (Brugada-Ramentol et al., in preparation). In addition, tactile discrimination is variable in non-psychotic subjects, an effect related to schizotypic differences among subjects (Lenzenweger, 2000). The existence of these basal differences in addition to the embodiment alterations observed in pathological conditions suggest the presence of genetic and molecular risk factors associated to embodiment defects. In addition, the heterogeneity of phenotypes observed in the different disorders points to specific components of embodiment that can be impaired, and to different molecular and neural pathways that can affect to these components.

## Possible embodiment-related molecular mechanisms

The variability across population observed in embodiment can be due to two specific sources: the genetic differences among individuals and the unique environmental or external factors that modulate the physiology of each subject. We have not found any reports on possible genetic factors altering the sense of embodiment in healthy individuals, but there are specific mutations in patients affected by frontotemporal dementia that correspond to self-perception alterations. For example, the presence of C9ORF72 or MAPT mutations correlate to different embodiment defects (Downey et al., 2014). In addition, we have previously mentioned that schizophrenic patients have an increased sense of agency (Garbarini et al., 2016) and several genetic factors have been associated to this condition using genome-wide association studies (GWAS) (Schizophrenia Working Group of the Psychiatric Genomics Consortium, 2014; Sekar et al., 2016). Interestingly, autism spectrum disorders, which also affect embodiment, share some of these genetic mutations in the following genes: APH1A, CNOTC, CSMDC, CUL3, CYPC7AC, CYP26BC, EPHX2, LRPC, MAPK3, MEF2C, MPP6, MYOC5A, NISCH, PBRMC, PRKDC, RIMS1, TSNAREC, WDR55, and ZNF80HA (Schizophrenia Working Group of the Psychiatric Genomics Consortium, 2014). Other mutations from neuropathological conditions (see Table 1 for a summary) can also be obtained, and, therefore, future studies could assess the overlapping mutations among these conditions that can be linked to specific alterations in embodiment, and their relationship to self-perception differences in healthy subjects.

From a physiological point of view, the diverse origins of embodiment variability could converge into similar outputs. For example, a genetic change or a pharmacological treatment might target to different molecules, but at a downstream level they might merge into a common pathway. As embodiment is a complex phenotype requiring several cognitive components, it is evident that multiple pathways might be involved. The use of neuronal imaging techniques like fMRI will be needed to assess how molecular differences can correlate with brain activation patterns, as some of these pathways will converge at the neural level, as shown for the oxytocin receptor epigenetic changes (Puglia et al., 2015). Nevertheless, in this article we only focus on the molecular pathways for two reasons: (i) the efficiency of state-of-the-art molecular techniques to obtain a complete picture of the physiological state of the subject at different biological levels, and (ii) the simplicity of these methods for scientists in fields other than molecular biology. In summary, our key point is that we can study the differences in embodiment looking for intermediate, molecular outputs instead of genetic and environmental differences, much more complex and uncontrollable. This studies could be complementary to the analyses of neural circuitry.

## Techniques for molecular profiling of embodiment

Embodiment is a complex phenotype in which several molecular pathways and neural circuits probably contribute to a proper self-representation. We thus expect the implication of several molecules and circuits at the same time in self-representation variability. This argues for the use of *-omic* techniques to obtain system-level results using a single sample from an individual. The combination of several of these *-omic* techniques will also increase the reliability of the markers obtained, reducing the amount of false positives (Ge et al., 2003).

We will describe three different classes of *-omic* techniques, depending on their functional level within the body. First, genomic techniques try to assemble the complete set of DNA of an organism. As every cell in the body has the same genome, individual samples can be obtained from simple procedures like either saliva or blood extraction. Massive DNA sequencing would be usually performed in an external facility, with a minimal sample preparation, and with a cost decreasing due to technical advances. Its main problem is the distance between DNA sequence and its actual cellular function, like neuronal activation. Therefore, the effect observed in cognitive phenotypes is usually very low, and thousands of subjects need to be studied in order to find significant associations with genetic factors (Sekar et al., 2016).

As a second level of *-omic* techniques, we include epigenomics and transcriptomics. These normally use sequencing as in genomics, so the complete set of chromatin (epigenetic) and expression (transcriptional) states of a sample, respectively, can be obtained. We can describe both results as regulatory profiles of an individual, because epigenomic and transcriptomic changes correspond to variations in the physiology of the subject. This is their main difference respect to genomics, resulting in a more functional level that can reflect significant correlations even in cognitive phenotypes. Their cost and simplicity is similar to genomics, but they also have a clear disadvantage: epigenetic and transcriptional changes are tissue-specific, so you need to either access brain samples or analyze the indirect changes produced in other tissues like blood or saliva. Nevertheless, efforts are made in order to assess neuroepigenetic changes using brain imaging (Yeh et al., 2013; Wey et al., 2016).

Finally, the third level of *-omic* techniques are proteomics and metabolomics. The set of proteins and metabolites, respectively, of a sample cannot be obtained by sequencing as in the former cases. The cost is usually higher, the protocols needed to prepare the samples are not as simple, and it is sometimes difficult to find an external facility that can analyze the samples. Nevertheless, proteomics and metabolomics have three strong advantages. They are regulated like in the case of epigenomics and transcriptomics, they are often secreted into fluids like blood or saliva, so we can use a simple extraction, and, in addition, proteins and metabolites are usually final outputs of the organism, so they are very close to the real function that we want to measure. In this case, some metabolomic approaches have deciphered brain neurophysiology and connectomics (Piomelli et al., 2007; Ivanisevic et al., 2014).

## Molecular-to-embodiment workflow

When studying the behavioral component of embodiment, there is an increasing need to rely more on physiological and implicit measures, and less on the use of explicit measures (e.g., self-report statements). In this case, molecular assessment could be used complementary to implicit behavioral measures of body ownership and sense of agency (i.e., proprioceptive drift, threat to virtual body or body-part, and intentional binding) and their changes related to embodying over a fake body part. They can also become complementary to the already existing imaging studies.

Previous studies has shown that people feel ownership over a virtual arm or body in experimental conditions that allow first-person perspective (Slater et al., 2010), shape and texture of the fake hand (Haans et al., 2008), congruent position of the hand (Tsakiris and Haggard, 2005), among others. Moreover, recently there has been an increasing use of virtual reality. For a potential experiment to study embodiment using molecular techniques, we would suggest taking advantage of virtual reality. This technique allows to create environments with ecological characteristics that resemble the real environment, while still being able to control for experimental variables (Tarr and Warren, 2002; Parsons, 2015). We propose that tissue samples (saliva or blood) could be extracted before exposure to the experiment of embodying a virtual arm; this will allow to study possible correlations with basal proteomic levels of each participant and the explicit self-report answers, implicit measures, and neuronal imaging patterns. Moreover, samples should also be extracted after exposure, allowing to compare changes in proteomic content before and after exposure to the experiment. For this end, we would suggest two experimental groups. The control group would be exposed to a virtual reality environment, measuring their basal capability to embody an external object as part of their own representation, without any modulation to decrease this embodiment. Moreover, a second group would be necessary, in which the embodiment over the virtual arm would be diminished using self-perception modulations. Finally, we would look for (i) molecular markers for the basal differences in the embodiment of the virtual arm in all participants; and (ii) molecular markers for the embodiment differences between groups with and without manipulation of self-perception, as a result of the manipulations that were applied to the virtual arm with the intention to reduce the embodiment over it. Furthermore, these embodiment differences could be assessed by explicit measures like self-reports or implicit techniques like brain activation pattern changes (Ehrsson et al., 2005; Petkova et al., 2011). Indubitably, we are aware that this kind of experiment would require a large number of subjects to have a significant quantity of data for each of the groups. Furthermore, we suggest that these studies could try to manipulate the sensory input and feedback in neuropathological conditions known to alter embodiment and to compare them to healthy controls.

Moreover, self-perception in healthy subjects could be altered in a way that resembles a specific neurological disorder. In this context, the use of -*omic* techniques to study the molecular profile of embodiment would benefit in understanding the effect of manipulations on the bodily self-representation. Additionally, it has been shown that the hormone oxytocin can modulate judgment over self-owned vs. other-owned objects (Wu et al., 2013). Therefore, we suggest to use the already proposed experiment combined with its treatment to improve sense of ownership and agency over the virtual arm. In this context, considering the virtual arm could be felt as an external object that is included into the self-representation. Therefore, the treatment with hormones like oxytocin should improve embodiment over these external objects. In addition, oxytocin treatments have been shown to alter the activity of specific brain areas using fMRI (Bethlehem et al., 2013). Finally, *-omic* techniques should help to assess the specific molecular changes in ownership and agency that are related to the neural changes after the administration of the hormone.

## Conclusion

In conclusion, the study of self-representation is a growing field in both healthy and clinical populations, due to the alterations that can be observed during pathological or experimental conditions. To this day, the use of imaging approaches is providing a greater insight to the study of embodiment, adding relevant brain activity information to behavioral assays. In addition to these, we propose that molecular profiling of healthy and clinical subjects could offer a new entry point to study embodiment. The combination of state-of-the-art techniques from different scientific fields, such as virtual reality, neuroimaging and *-omic* methods, is a promising approach to understand self-representation from a systems perspective.

## Author contributions

GGdP, ÁCR, and VBR wrote the manuscript. ÁCR and VBR developed the idea of the manuscript.

## Funding

We acknowledge funding from the Fundaçao para a Ciência e Tecnologia (PTDC/NEU-SCC/0948/2014) and the Champalimaud Research (Portugal) to GGdP, from a FEBS long-term postdoctoral fellowship to ÁCR, and from Fundaçao para a Ciência e Tecnologia Ph.D. fellowship to VBR SFRH/BD/52201/2013).

### Conflict of interest statement

The authors declare that the research was conducted in the absence of any commercial or financial relationships that could be construed as a potential conflict of interest.
